# Supplementary material for: A conformation-based intra-molecular initiation factor identified in the flavivirus RNA-dependent RNA polymerase
Source: PLoS Pathog. 2020 May 1;16(5):e1008484. doi: 10.1371/journal.ppat.1008484 (PMC7219791; doi:10.1371/journal.ppat.1008484)
Supplement: Attachment — Submitted filename: point_by_point_responses.docx [file ppat.1008484.s002.docx]

**Point-by-point responses to reviewers’ comments for**

**manuscript tracking #: PPATHOGENS-D-19-02325**

*A conformation-based intra-molecular initiation factor identified in the flavivirus RNA-dependent RNA polymerase*

by Jiqin Wu, Han-Qing Ye, Qiu-Yan Zhang, Guoliang Lu, Bo Zhang, Peng Gong

**(Our responses are shown in boldface text with grey background)**

Reviewer Comments (if any, and for reference):
Reviewer's Responses to Questions

**Part I - Summary**
Please use this section to discuss strengths/weaknesses of study, novelty/significance, general execution and scholarship.

Reviewer #1: In this manuscript, Wu et al. report the crystal structures Dengue virus type 2 (DENV2) NS5 and show that it exists in two distinct conformations representing both of the previously reported structures of Japanese encephalitis virus (JEV) and DENV3 NS5. These are defined as JEV-mode and DENV3-mode due to closeness of these DENV2 structures to the JEV and DENV3 NS5 structures. Moreover, the authors present additional data by mutational analysis to distinguish the functional differences between the two conformations of DENV2 NS5. They performed in vitro polymerase assays with wild-type (WT) and site-specific NS5 mutants to distinguish the initiation and elongation steps of RNA synthesis as well as cell-based replication using immunofluorescence assays. The authors conclude that the methyltransferase (MTase) of NS5 serves as a unique initiation factor only through NS5 conformation in the JEV-mode. But no MTase assays were performed. Overall, the crystallographic work is well-done. However, their biochemical and virological assays raise several questions. The functional roles of these two conformational states of DENV2 NS5 in viral replication remains unclear. Moreover, another group reported recently that DENV2 NS5 exists in two distinct conformational states (El Sahili, Soh, Schiltz, Gharbi-Ayachi, She, Shi, Lim, Lescar, 2020. J. Virol. 94: e01294-19). These authors in El Sahili et al. provide convincing evidence that it is the inter-domain linker between the MTase and POL domains the functional determinant of the two conformational states of DENV2 NS5.

**In the Sahili et al. paper, only one DENV2 NS5 conformation was reported, corresponding to the JEV-mode conformation described in our study. The functional study in this paper was primary based on MTase-RdRP linker substitutions using linker sequences from other DENV serotypes or other flaviviruses. We correctly cited this structure in our introduction and have added related description in the revised manuscript.**

Reviewer #2: The flaviviral NS5 proteins contain a methyltransferase (MTase) domain followed by an RNA-dependent RNA polymerase (RdRP) domain, both of which have essential functions during viral replication. Structures of the individual domains have been known for over a decade and biochemical approaches have been applied to studying the seemingly independent activities of these domains; this has worked moderately well for the MTase, but not so well for the RdRP due to the presence of a priming loop on the thumb domain that limits RNA binding and has precluded detailed biochemical studies of elongation kinetics the solving of an active elongation complex structure. Over the past few years that have several structure of full length NS5 solved from multiple flaviviruses (JEV, ZIKV, DENV3), and interestingly these have shown different interactions between the MTase and RdRP domains. However, it has not yet been clear if the observed inter-domain interactions are biologically relevant or due to crystal packing effects.
The work presented in this manuscript provides a thorough study of MTase-RdRP interactions using structural biology, biochemistry, and virus replication data to address functional roles of the flaviviral methyltransferase (MTase) during the initiation of replication by the RdRP domain. Two new structures of DENV2 NS5 are solved that capture domain orientations which are intermediate between those solved previously for DENV3 and JEV. Structure-based mutagenesis is then used to show that one specific mode of MTase-RdRP interaction (the JEV mode) is important for both the growth of infectious virus and for in vitro initiation by the RdRP domain. These new results provide key insights into understanding the molecular mechanisms whereby flaviviral NS5 initiates replication.
Using initiation of a pGG dinucleotide to form pGGA or to extent to a longer 9mer when UTP is also added, the authors use mutations of the show that formation of both products is diminished upon mutation of the JEV-mode interface but not the DENV3 interface, indicating that the JEV-mode is the active form. Furthermore, the researchers demonstrate that mutating the MTase interaction only affects RdRP initiation to form short products (pGG to pGGA), and does not affect on the elongation of a longer 9mer product to a 10mer. This latter elongation step is demonstrated to be very rapidly done by a complex that is stable in high salt, showing that the authors have assembled authentic stalled elongation complexes form a flaviviral polymerase. This is a major breakthrough that sets the stage for future studies of both RdRP biochemistry and elongation complex structure.
The core experiments and conclusions are solid and well presented in the manuscript and there are no major concerns with the interpretation of the results. As usual from Gong lab, the illustrations are excellent and the polymerase biochemistry is well done with rigorous interpretations, all the more notable in this work because of the extensive steps taken to normalize Stains-All data across multiple gels. However, there are a few points where additional clarification or grammar corrections would be appreciated, as outlined below.

**We have made grammar corrections as suggested.**

Line 146-149: It may be worth discussing that the ring finger and part of index finger are not resolved in many of the early flaviviral RdRP domain structures (2hcn, 2hfz, 2j7u, 2j7w… see the flaviviral structures at DOI: 10.5281/zenodo.3361874). Might this be because the MTase domain is needed to stabilize the fingers folding, which could have big effects on RNA binding and initiation?

**We agree with this comment, and have added related descriptions in the results section.**

The section from lines 162-167 describing SAH binding site is weak and the evidence for an “allosteric” site sounds somewhat circumstantial. Is there any experimental evidence for SAH binding being important, or is it perhaps a crystallization artifact? The section should either be strengthened with experimental data or references, or the idea of “allosteric” should be removed and the observed SAH density instead be described as an interesting structural observation that merits further biochemical validation. Also, was SAH added to the crystallization conditions, or did this SAH co-purify with the protein much as the SAH bound to the MTase domain does? Is there full occupancy of this “allosteric” site and of the native MTase site? Is there any (weak) anomalous density from the sulfur that would definitively identify the SAH?
**We agree with this reviewer that this SAH binding site cannot be described as "allosteric", and have used "secondary binding site " instead. We have used "putative" to describe this SAH binding site since we have not been able to collect evidences such as anomalous signal of sulfur and SAH is not present in our crystallization buffer. Related descriptions have been provided in the results section.**

Please explicitly state the actual concentration of polymerase, RNA template and pGG primer used in the biochemical experiments. These are not stated anywhere in the manuscript, where only mole ratios are listed.

**The actual concentrations of polymerase and RNA have been provided in the revised version.**

**Part II – Major Issues: Key Experiments Required for Acceptance**
Please use this section to detail the key new experiments or modifications of existing experiments that should be absolutely required to validate study conclusions.

Generally, there should be no more than 3 such required experiments or major modifications for a "Major Revision" recommendation. If more than 3 experiments are necessary to validate the study conclusions, then you are encouraged to recommend "Reject".

Reviewer #1: In this manuscript, Wu et al. report the crystal structures Dengue virus type 2 (DENV2) NS5 and show that it exists in two distinct conformations representing both of the previously reported structures of Japanese encephalitis virus (JEV) and DENV3 NS5. These are defined as JEV-mode and DENV3-mode due to closeness of these DENV2 structures to the JEV and DENV3 NS5 structures. Moreover, the authors present additional data by mutational analysis to distinguish the functional differences between the two conformations of DENV2 NS5. They performed in vitro polymerase assays with wild-type (WT) and site-specific NS5 mutants to distinguish the initiation and elongation steps of RNA synthesis as well as cell-based replication using immunofluorescence assays. The authors conclude that the methyltransferase (MTase) of NS5 serves as a unique initiation factor only through NS5 conformation in the JEV-mode. But no MTase assays were performed. Overall, the crystallographic work is well-done. However, their biochemical and virological assays raise several questions. The functional roles of these two conformational states of DENV2 NS5 in viral replication remains unclear. Moreover, another group reported recently that DENV2 NS5 exists in two distinct conformational states (El Sahili, Soh, Schiltz, Gharbi-Ayachi, She, Shi, Lim, Lescar, 2020. J. Virol. 94: e01294-19). These authors in El Sahili et al. provide convincing evidence that it is the inter-domain linker between the MTase and POL domains the functional determinant of the two conformational states of DENV2 NS5.
**We are considering the suggested MTase characterization in future studies. The relevance of the MTase-RdRP linker to the conformational states has been discussed in the revised version with the Sahili et al. work specifically mentioned. As mentioned above in response to this reviewer, this paper only report one conformation of DENV2 NS5 (PDB entry 5ZQK).**
Specific comments
1. Line 71, p. 4: This study focuses only on the polymerase activity of NS5 although the interdomain interaction between MTase and POL domains could impact on both enzyme activities. Why the authors did not perform any MTase assays to study the impact of the two conformations of DENV2 NS5?

**As mentioned above, we are considering the suggested MTase characterization in future studies. Wherever necessary, we have discussed that the intra-molecular interactions between MTase and RdRP could also affect the MTase activities.**

2. Lines 99-101, p. 5: “This statement is not correct. Once initiated de novo, RdRp can continue RNA synthesis until the template is copied. This has been demonstrated for dengue virus type 2 RdRp in many studies using subgenomic RNA templates containing 5’- and 3’-terminal regions and conserved cis-active elements that have been shown to be important for both initiation and elongation steps (see refs. Ackermann and Padmanabhan, 2001; Niyomrattanakit et al. 2010). The statement on line 101 is not accurate because in one study, processivity of RdRp was demonstrated by including heparin after initiation complex is formed to block re-initiation events (Nomaguchi et al. 2003). The statement on line 102 needs to modified to indicate that both Mg++ and Mn++ were used (in refs. 18, 24 and 25 cited in this study).
**Most of the subgenomic RNA-based assays, including all three references mentioned by this reviewer, used radio-labelled NTP to detect the RNA products but without reporting the amount of the product relative to the RNA template. Therefore, it is difficult to judge whether the full-length product was generated by the majority of the polymerases, or from only a small fraction of them. Nevertheless, we modified our descriptions with the comments considered.**

3. The effects of mutations on MTase activity were not studied whereas these were examined in ref. 18 cited in this study.

**As mentioned above, we are considering the suggested MTase characterization in future studies.**
4. Lines 204, 208, 264: There is no Fig. 3E included in the manuscript.

**Thanks for pointing this out. We mistakenly used an incorrect version of this figure at our initial submission. The correct version of this figure (including panel E) has been provided.**
5. Lines 235-239 and lines 239-242: Please cite the ref(s) here; 12 or 33?.

**Both references have been specifically cited.**
6. One concern with this study is the use of artificial GG dinucleotide primer and template for RdRp initiation and elongation assays. The authors’ definition of de novo initiation in mosquito-borne RdRp assays is unconventional and may lead to wrong conclusions regarding the mutational effects in RdRp activities. For example, the catalytic rate constant could not be accurately determined under their conditions (lines 252-257).
**Dinucleotide has been used in multiple *de novo* polymerase systems (including DNA-dependent RNA polymerase, the Jin et al. HCV study cited in our manuscript, and in influenza virus polymerase, etc.) to mimic the *bona fide* *de novo* initiation. The fact that the conversion of GG to the trinucleotide GGA had multiple turnover behavior indicates that this process is consistent with unstable initiation. Once converted to the 9-mer product, the single turnover behavior shows the evidence of stable elongation complex formation. We have added description and citations for dinucleotide-based initiation accordingly.**

Reviewer #2: None

**Part III – Minor Issues: Editorial and Data Presentation Modifications**
Please use this section for editorial suggestions as well as relatively minor modifications of existing data that would enhance clarity.

Reviewer #1: Minor Comments:
1. Line 144: Which DENV3 structures the authors are referring to here? It would be helpful if the authors label the motif G in Fig. 1.

**In line 144, we meant both DENV3 structure and both citations were provided. We have added the label for motif G in Figure 1 as suggested. The PDB entry used for the DENV3 NS5 model has been provided in the figure legend of Figure 1.**
2. Lines 160-162: The SAH binding site has been observed and labeled in Klema et al. (ref. 21).

**In the Klema et al. structure, SAH molecules are bound in the SAM binding pocket but not in a secondary site.**
3. Define “pt” ( line 229) andNS5B NTD (line 403).
**"pt" (post transfection) was defined in the same paragraph. NS5B NTD was defined one paragraph ahead.**

Reviewer #2: Line 40: calling differences in prior structures “drastically different global folds” is perhaps an overstatement since the effects are really at the level of relative domain orientations and not protein folding.

**We have used "apparently" to replace "drastically".**
Line 51: remove “about” as it redundant with giving a range of 10-11 kb for genome length.
**Modified as suggested.**
Line 71: change are to is
**Modified as suggested.**
Line 72: make evidences singular
**Modified as suggested.**
Line 76: typo in hereafter
**We tend to use "hereinafter" here.**
Line 86: in discussing interface areas, please explicitly state if the values presented reflect total buried solvent accessible surface from both sides of the interface, or from only one side of the interface. Also on lines 138, 157 and others.
**We have made it clear that both sides of the interface were accounted.**
Line 115: Consider deleting “apparently” as the data for this are convincing.
**Modified as suggested.**
Line 120: change in to to.
**Modified as suggested.**
Line 127: arrange should be in past tense (arranged).
**Modified as suggested.**
Line 301: The same high-salt challenge studies are also used in studies of picornaviral polymerases, where they led to the crystallization of elongation complexes.
**We have added the citation of a related poliovirus polymerase work (Hobdey et al 2010).**
Line 312-316: What really needs to the optimized here is the amount of material loaded on the gel, not necessarily what was used in the reactions, which could be done at higher concentrations and then diluted prior to gel loading (a minor conceptual point, no need to edit the text).
**We thank this reviewer for this valuable suggestion.**
Line 316: Change 3-fold to “3-fold higher”
**We have used "three times the values of the WT and M_67A/68A mutant" instead.**
Line 375: I do not agree with the statement that the global structural organization is quite different from flaviviral polymerases versus others. Structure comparisons have shown the core polymerase fold and structure to be very similar across all viral polymerases, and certainly among positive strand RNA viruses, with few insertions or topology changes within the core fold. There are added domains, such as the N-terminal MTase and NiRAN domains or C-terminal membrane anchors, but these are additions to the protein and not a reorganization of the protein fold as implied by the manuscript as written.
**We understand the concern by this reviewer and have rephrased our description using "the overall structure" instead of "the global structure organization".**
Line 394: I do believe there is convincing evidence that the N-terminal region of nsp9 is a nucleotidylating NiRAN domain.
**We thank this reviewer for pointing this out, and have added the description of the nucleotidyltransferase (NiRAN) information and the related citations.**
Line 518: Include actual concentrations of macromolecules used in the experiments.
**Concentrations have been provided as suggested.**
Figure 3: Are the stereo panels in A and B flipped right to left? Stereo viewing of these images does not seem to work correctly.
**Panel A stereo images are arranged as standard wall-eyed mode, while panel B is not stereo. We have modified the related figure legends to clarify this.**
Figure 5: In panel D, why is the 9mer STD band higher than the 9mer product band from the polymerase? Effect of the 5’ phosphate from pGG primer, perhaps?
**This reviewer's judgment is correct, and the 5’-phosphate indeed affected the migration. We have added related description in the figure legend.**
Line 816: Consider changing to “…in the multiple turnover P3 formation and single turnover P9 formation assays” so as to explain the vast difference in band intensities in the figure legend itself (in addition to the comments already in the text of the manuscript).

**This is a good suggestion and we have modified the figure legend accordingly.**
Line 833: Technically speaking, the experiment in really testing inactivation of the elongation complex and not the dissociation rate of the RNA, although that is probably a valid interpretation in this case.

**We agree with this comment that inactivation may be more appropriate. This is a good suggestion and we have changed the dissociation rate constant (*k_dis_*) to inactivation rate constant (*k_ina_*) in the main text and in Figure 6D and related figure legend.**
Figure 7: In some cases, linear curve fits to determine initial rates are being applied to data that have clear curvature (panel E-200uM, panel F-50 & 100 uM, panel G-R3). This is a minor point.

**We understand the concern for the fitting itself. The linear fitting was chosen with the assumption that the P3 formation rate is constant if the ATP concentration does not change much over time, which was the case in our experimental settings.**
